# Molecular Design, Structural Analysis and Antifungal Activity of Derivatives of Peptide CGA-N46

**DOI:** 10.1007/s12539-016-0163-x

**Published:** 2016-05-10

**Authors:** Rui-Fang Li, Zhi-Fang Lu, Ya-Nan Sun, Shi-Hua Chen, Yan-Jie Yi, Hui-Ru Zhang, Shuo-Ye Yang, Guang-Hai Yu, Liang Huang, Chao-Nan Li

**Affiliations:** College of Biological Engineering, Henan University of Technology, Zhengzhou, 450001 China

**Keywords:** Antifungal peptides, CGA-N46, Derived peptide, Bioinformatic analysis, Molecular design, Structural analysis, Antifungal activity, Hemolytic activity

## Abstract

Chromogranin A (CGA)-N46, a derived peptide of human chromogranin A, has antifungal activity. To further research the active domain of CGA-N46, a series of derivatives were designed by successively deleting amino acid from both terminus of CGA-N46, and the amino acid sequence of each derivative was analyzed by bioinformatic software. Based on the predicted physicochemical properties of the peptides, including half-life time in mammalian reticulocytes (in vitro), yeast (in vivo) and *E. coli* (in vivo), instability index, aliphatic index and grand average of hydropathicity (GRAVY), the secondary structure, net charge, the distribution of hydrophobic residues and hydrophilic residues, the final derivatives CGA-N15, CGA-N16, CGA-N12 and CGA-N8 were synthesized by solid-phase peptide synthesis. The results of bioinformatic analysis showed that CGA-N46 and its derivatives were α-helix, neutral or weak positive charge, hydrophilic, and CGA-N12 and CGA-N8 were more stable than the other derivatives. The results of circular dichroism confirmed that CGA-N46 and its derived peptides displayed α-helical structure in an aqueous solution and 30 mM sodium dodecylsulfate, but α-helical contents decreased in hydrophobic lipid vesicles. CGA-N15, CGA-N16, CGA-N12 and CGA-N8 had higher antifungal activities than their mother peptide CGA-N46. Among of the derived peptides, CGA-N12 showed the least hemolytic activity. In conclusion, we have successfully identified the active domain of CGA-N46 with strong antifungal activity and weak hemolytic activity, which provides the possibility to develop a new class of antibiotics.

## Introduction

Over the past decades, the widespread use of antibiotics has led to the rapid emergence of antibiotic-resistant bacteria [1, 2]. Antimicrobial peptides (AMPs) exhibit broad-spectrum activity against bacteria, fungi, viruses, parasites and even cancer cells. AMPs were recently determined to be potential candidates of conventional antibiotics for treating drug-resistant bacterial infections [3, 4]. These peptides have been isolated from many natural sources including microorganism, insects, animals and plants [5, 6]. As of July 2015, the current AMP database contains over 2500 AMPs (http://aps.unmc.edu/AP/main.php) [7].

Chromogranin A is a soluble protein which exists in endocrine cells and neurons [8, 9]. The endogenous chromogranin A-derived peptides, such as vasostatin-I and catestatin, have been described to be natural defensive barriers for human body [10, 11]. Vasostatin-I (CGA1-76), an N-terminal fragment of chromogranin A, is able to kill a large variety of fungi and yeast cells in micromolar range [12]. Lugardon group synthesized several derived CGA N-terminal fragments. The results of their antifungal research indicated that the shortest active peptide corresponded to the sequence Arg47–Leu66, and named chromofungin. CGA-N46, a novel antifungal peptide containing the 31st to 76th amino acids of human chromogranin A, showed strong anti-*Candida* activity. Li et al. [13] successfully expressed CGA-N46 in engineered *Bacillus subtilis* strain DB1342 and optimized the expression and purification protocol. However, the yield and the purity of peptide CGA-N46 still could not meet the demand of research.

Solid-phase peptide synthesis (SPPS) is another method to prepare peptide. Compared with genetic engineering expression, SPPS has many advantages including efficient synthesis, easy purification and high purity. However, the difficulty in maintaining the correct structure and function for synthesized peptides increase with the lengthening of peptides [14].

In this report, in order to further study the antifungal active domain, CGA-N46 was analyzed by bioinformatics software. The potential antifungal-derived fragments were designed. The structures and biological activities of the designed derivatives were further investigated to find the candidates with strong antifungal activities and bio-safety.

## Methods

### Microorganisms and Reagents

*Candida glabrata* (ATCC-90525), *Candida parapsilosis* (ATCC-20224), *Candida krusei* (ATCC-6258), *Candida tropicalis* (ATCC-20240), *Candida albicans* (ATCC-2048) were supplied by the Chinese Academy of Medical Sciences.

CGA-N46 and its derivatives were synthesized by solid-phase peptide synthesis method. Peptide purification was performed using high-performance liquid chromatography (HPLC). The mass of each peptide was confirmed via mass spectrometry. Final purity of the peptides was determined to be 90 % by analytical HPLC.

### Physicochemical Properties Analysis

The structural prediction software ProtParam tool in bioinformatics website ExPASy (http://www.expasy.ch/tools/) was used to predict the physicochemical properties of the peptides, including molecular weight, isoionic point (PI), half-life time in mammalian reticulocytes (in vitro), yeast (in vivo) and *E. coli* (in vivo), instability index, aliphatic index and GRAVY. Peptide was predicted to be stable when instability index was less than 40. Otherwise, peptide was assumed to be unstable. The heat stability of peptide was indicated by its aliphatic index. The higher aliphatic index means higher heat stability. The hydrophilicity and hydrophobicity of peptide were predicted by GRAVY. The peptide was hydrophobic when the GRAVY value was plus; otherwise, it was hydrophilic.

### Amino Acid Distribution Analysis

The Helical Wheel Projections software in web (http://rzlab.ucr.edu/scripts/wheel/wheel.cgi) was used to predict the distribution of the hydrophobic and hydrophilic residues of the derived peptides.

### Preparation of Small Unilamellar Lipid Vesicles

Small unilamellar lipid vesicles (SUV) were prepared according to the method [15, 16] with modification. One hundred mg of phosphatidylcholine (PC): phosphatidylglycerol (PG) (3:1 weight ratio) dissolved in chloroform was dried by rotating evaporation under a vacuum to form a lipid film on the round bottom glass bottle wall. The obtained lipid film, composed of 75 mg of PC and 25 mg of PG, was rehydrated with 10 ml of 20 mM potassium phosphate buffer (pH 7.0) to the final lipid concentration of 10 mg mL^−1^. SUVs were prepared by ultrasonic processing the sample with pulses ‘15 s on/45 s off’ for 10 min at 4 °C and an input power of 40 W until the suspension was transparent. The peptides were added to SUVs at concentrations of 0.25 mg mL^−1^ and incubated at room temperature for at least 30 min prior to the measurements.

### Circular Dichroism Assay

Circular dichroism (CD) spectroscopy was performed using a MOS-500 spectropolarimeter (Bio-Logic, MOS-500, France) with the method according to the report [17] with modest modification. CD spectra of the peptides were recorded between 190 and 240 nm of scanning spectrum at 1 nm intervals at 25 °C with a scanning speed of 100 nm min^−1^, 2 s of response time, 1.0 nm of step size.

Peptides with a constant concentration of 0.25 mg mL^−1^ were prepared in three different solvents, 20 mM phosphate buffer, pH 7.4 (mimicking the aqueous environment), 30 mM sodium dodecyl sulfate (SDS, mimicking the negatively charged environment of microbial membrane) and SUVs (mimicking the hydrophobic environment of the microbial membrane). The samples were loaded in a rectangular quartz cuvette with a path length of 1 mm. The spectra of three consecutive scans were averaged and corrected by subtracting the solvent/buffer spectra. The mean residue molar ellipticities were calculated using the equation *θ* = (*θ*_obs_·1000)/(*c*·*l*·*n*) [18], where *θ*_obs_ is the ellipticity in millidegrees; *c* is the peptide concentration in mole l^−1^; *l* is the optical path length of the cuvette in centimeters; and *n* is the number of peptide residues.

The CD data of CGA-N46 and its derivatives were analyzed by CDPro software package including SELCON, CONTIN and CDSSTR to study the conformational changes in CGA-N46 and its derivatives in the aqueous, SDS and SUVs environments.

### Antifungal Assays

Minimum inhibitory concentrations (MICs) of CGA-N46 and its derivatives against fungi were measured according to a modified version of broth microdilution method of the Clinical and Laboratory Standards Institute (CLSI) [19]. Fungi cell’s viability was assessed based on the reduction of 3-[4,5-dimethylthiazolyl]-2,5-diphenyltetrazolium bromide (MTT) into formazan dye by active mitochondria [20]. Briefly, peptides were serially twofold diluted in 20 mM PBS (pH 6.0) to a final concentration between 2 mg mL^−1^ and 3.9 µg mL^−1^. Subsequently, 100 μL samples was dispensed into the wells of a 96-U-shaped-well plate, and each was mixed with 100 μL of the 2 × inoculum suspension (1–5 × 10^4^ CFU mL^−1^) of a log-phase fungal culture in Sabouraud (SD) broth. Strains without treatment with peptides were used as negative control. The cultures were incubated at 30 °C without agitation for 16 h. 10 μL of MTT solution (5 mg mL^−1^ MTT in PBS) was added to each well, and the plate was further incubated for 4 h. After rinsed, 100 μL of dimethylsulfoxide (DMSO) was added to dissolve the MTT formazan crystals. The inhibition of growth was determined by measuring the absorbance at 570 nm with a microplate reader. The relative cellular activity was calculated according to the following formula:

$$\begin{aligned} & {\text{cell survival inhibition rate (\% of control)}} \\ & \quad {\text{ = [(OD}}_{ 5 7 0} {\text{ of the negative sample}} - {\text{OD}}_{ 5 7 0} {\text{ of the treated sample) / OD}}_{ 5 7 0} {\text{ of the negative control]}} \times 1 0 0\;{\text{\%}} .\\ \end{aligned}$$

And the MIC was defined as the lowest peptide concentration that completely inhibited fungal growth.

Each test was performed in triplicate, and the data were expressed as the mean ± SE.

### Hemolytic Assay

Hemolytic activity was tested according to the method [21]. Briefly, peptides were serially twofold diluted using PBS in 96-well plates to give a volume of 100 μL of the sample solution in each well, and the final concentration of peptides ranged between 2.0 mg mL^−1^ and 3.9 μg mL^−1^. Human red blood cells (RBCs) from a healthy volunteer were diluted to a concentration of 2 % in PBS. 100 μL of the RBC suspension was added to each well, and the reactions were incubated at 37 °C for 60 min, followed by 150 μL supernatant being transferred to a new 96-well plate (U-shaped well). The release of hemoglobin was determined by measuring the absorbance of the supernatant at 570 nm. RBCs in PBS and 0.1 % (v/v) Triton-X100 were used as the negative and positive controls, respectively. The percentage of hemolysis was calculated using the following formula:

$$\begin{aligned} {\text{Hemolysis rate }}\left( \% \right) & = [({\text{OD}}_{570} {\text{of the treated sample}} - {\text{OD}}_{570} {\text{of the negative control}}) \\ & \quad /\left( {{\text{OD}}_{570} {\text{of the positive control}} - {\text{OD}}_{570} {\text{of the negative control}}} \right)] \times 100\;\% . \\ \end{aligned}$$

Each test was performed in triplicate, and the data were expressed as the mean ± SE.

### Statistical Analysis

Experimental data were analyzed using the PASW statistical 18 (SPSS, Inc., Chicago, IL, USA) to perform one-way analysis of variance followed by least significant difference and Duncan’s tests. The results are reported as mean ± standard error of the mean (SEM). Differences between treatment groups and the control group were considered to be statistically significant at *p* < 0.05 and extremely significant at *p* < 0.01.

## Results

### Peptide Design and Predicted Physicochemical Properties

Derived peptides were obtained by successively deleting amino acid from both ends of CGA-N46. The amino acid sequences of the derived peptides were analyzed by software ProtParam tool. The physicochemical properties of CGA-N46 and its derived peptides were predicted and compared. Four potential peptides with long half-life time and high stability were obtained, and were named as CGA-N15, CGA-N16, CGA-N12 and CGA-N8. The amino acid sequences are given in Table 1. CGA-N15 is the middle fragment of CGA-N46 with 15 amino acids. CGA-N16 is the C-terminal fragment of CGA-N46. CGA-N12 is a derived fragment of CGA-N16, and CGA-N8 is a derived peptide of CGA-N12.

**Table 1:** Amino acid sequences of CGA-N46 and its derived peptides

Peptide	Amino acid sequence
CGA-N46	NH_2_–PMPVSQECFETLRGHERILSILRHQNLLKELQDLALQGAKERAHQQ–COOH
CGA-N15	NH_2_–ERILSILRHQNLLKE–COOH
CGA-N16	NH_2_–LQDLALQGAKERAHQQ–COOH
CGA-N12	NH_2_–ALQGAKERAHQQ–COOH
CGA-N8	NH_2_–GAKERAHQ–COOH

As given in Table 2, all the derived peptides were near neutral or weak alkaline and hydrophilic. Among of them, CGA-N12 and CGA-N8 had longer half-life time and were more stable than CGA-N15 and CGA-N16. Compared with CGA-N8, the heat stability of CGA-N12 was higher.

**Table 2:** Physicochemical properties of four peptides derived from CGA-N46

Peptide	Molecular	PI	GRAVY	Half-life			Instability index	Aliphatic index
				Mammalian reticulocytes	Yeast	*E. coli*		
CGA-N46	5363.1	7.38	−0.674	>20 h	>20 h	Unpredicted	75.91	97.16
CGA-N15	1862.2	8.85	−0.447	1 h	30 min	>10 h	55.03	156.0
CGA-N16	1806.0	6.75	−1.012	5.5 h	3 min	2 min	91.88	91.88
CGA-N12	1336.4	8.8	−1.400	4.4 h	>20 h	>10 h	38.23	57.5
CGA-N8	895.9	8.75	−1.925	30 h	>20 h	>10 h	−12.48	25

### Amino Acid Distribution of CGA-N46 and Its Derivatives

**Fig. 1:**
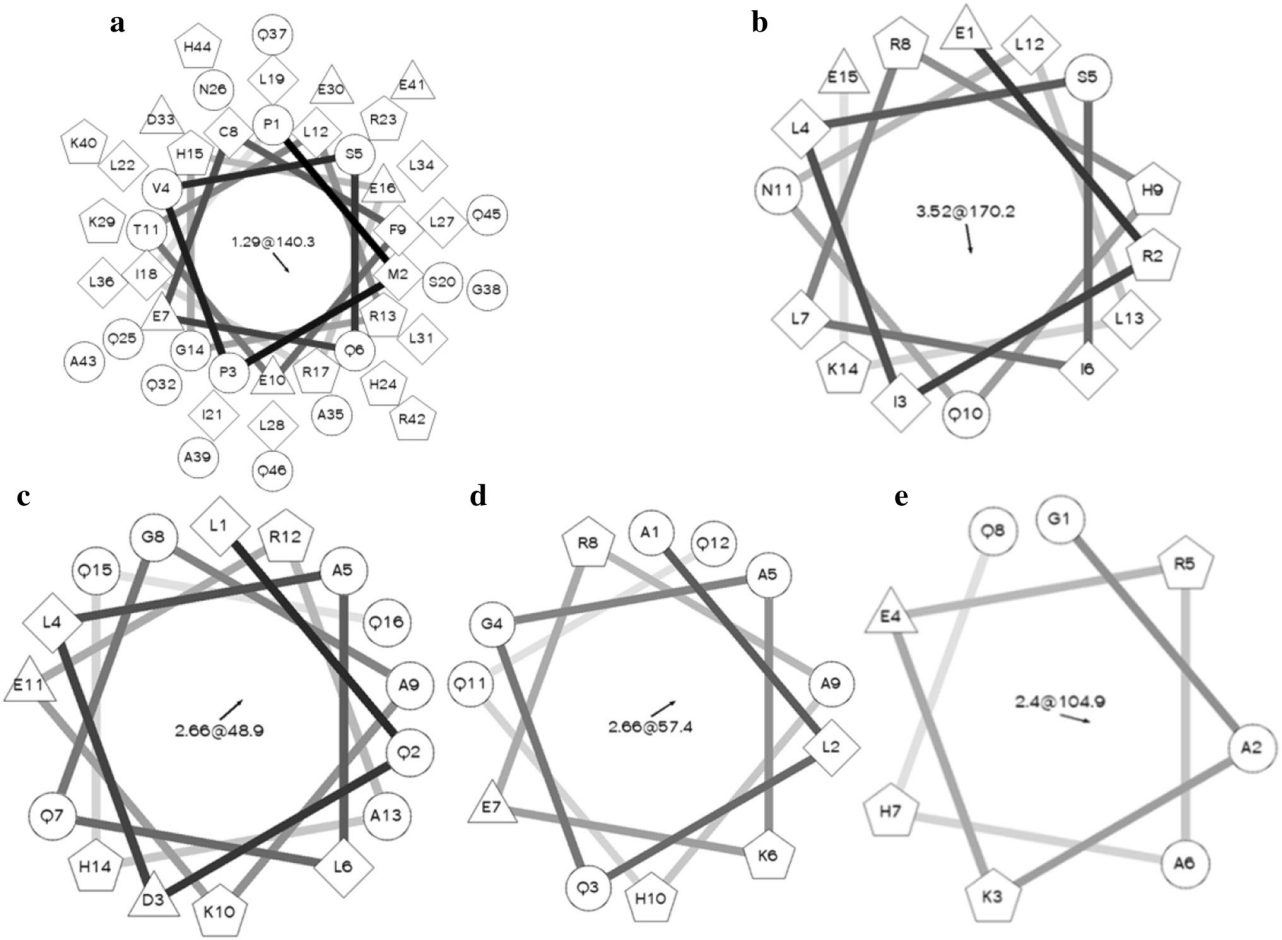
Predicted amino acid residues distribution of CGA-N46 and its derived peptides. **a** CGA-N46, **b** CGA-N15, **c** CGA-N16, **d** CGA-N12, **e** CGA-N8. *Note* Hydrophilic residues present as *circles*, hydrophobic residues as *diamonds*, potentially negatively charged as *triangles* and potentially positively charged as *pentagons*. Figure has been produced by Helical Wheel Projections and Photoshop

The amino acid sequences of the derived peptides were analyzed by software the Helical Wheel Projections. The amino acid distribution of CGA-N46 derived peptides were predicted as shown in Fig. 1. The results in Fig. 1 indicated that there were no typical hydrophilic face and hydrophobic face in the structure of peptide CGA-N46 and its derivatives. The hydrophobic residues, hydrophilic residues and the residues with positive or negative charges scattered randomly.

**Fig. 2:**
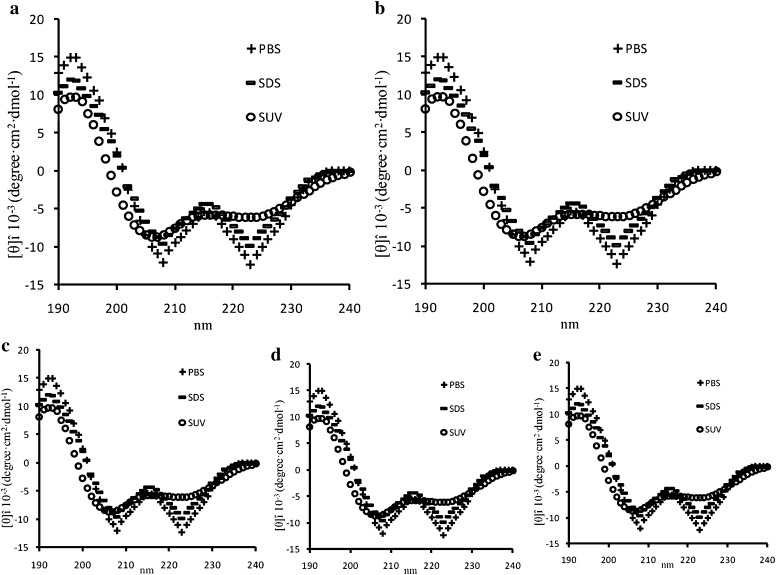
CD spectra of CGA-N46 and its derived peptides in different solution. **a** CGA-N46, **b** CGA-N15, **c** CGA-N16, **d** CGA-N12, **e** CGA-N8. Figure has been produced by Excel and Adobe Illustrator CS6

CD spectroscopy was performed to research the secondary structure of CGA-N46 and its derivatives peptides in aqueous environment, SDS and SUVs. The CD spectra were analyzed and the mean residue ellipticity is shown in Fig. 2. CGA-N46 and its derived peptides displayed a typical of α-helical conformation with characteristic double minima at 208 and 222 nm [22, 23]. Interestingly, an increase in the mean residue ellipticity at 208 and 222 nm was observed at the presence of SUVs. The CD spectra difference suggested that the secondary structural alterations of CGA-N46 and its derivatives were promoted when they interacted with cell membranes.

To examine the conformational percentage in the absence and presence of SUVs, the CD data of CGA-N46 and its derivatives were analyzed by CDPro software package including SELCON, CONTIN and CDSSTR. The percentage of secondary structure elements for CGA-N46 and its derived peptides (Table 3) demonstrated that the spectra of CGA-N46 resembled the spectra of its derived peptides in the absence and presence of SUVs; however, their intensity of α-helical in SUVs is somewhat lower than that in aqueous conditions, which indicated that hydrophobicity might reduce the content of α-helix and promote other folding conformation of CGA-N46 and its derivatives. This result is different from some related reports [24, 27, 28].

**Table 3:** Percentages of the secondary structural elements of CGA-N46 and its derived peptides

	α-Helix (%)	β-Sheet (%)	β-Turn (%)	Random (%)
CGA-N46				
H_2_O	79.57	15.79	3.54	1.1
SDS	80.32	14.96	2.32	2.4
SUV	38.74	25.96	22.79	12.51
CGA-N15				
H_2_O	82.69	10.89	3.65	2.77
SDS	83.41	11.25	3.82	1.52
SUV	41.53	30.47	13.96	14.04
CGA-N16				
H_2_O	83.67	11.16	3.23	1.94
SDS	83.33	11.43	3.36	1.88
SUV	40.91	35.67	12.59	10.83
CGA-N12				
H_2_O	88.36	8.64	1.56	1.44
SDS	87.91	9.53	1.38	1.18
SUV	43.21	32.09	15.39	9.31
CGA-N8				
H_2_O	87.79	8.56	1.34	2.13
SDS	84.89	9.87	1.79	3.45
SUV	43.76	34.27	13.86	8.11

### Antifungal and Hemolytic Activities of CGA-N46 and Its Derived Peptides

The antifungal activity of CGA-N46 and its derived peptides against several *Candida spp*. (Table 4) was used as an indicator of the peptide’s anti-*Candida* activity. CGA-N46 and its derived peptides were active against all of the tested yeasts (MICs, less than 0.5 mM). But interestingly, different yeast exhibited various greatest sensitivity for different peptides: *C. krusei* (MIC, 0.37 mM) for CGA-N46, *C. tropicalis* (MIC, 0.073 mM) for CGA-N15, *C. glabrata* (MIC, 0.28 mM) for CGA-N16, *C. tropicalis* (MIC, 0.075 mM) for CGA-N12, and *C. krusei and C. albicans* (MIC, 0.24 mM) for CGA-N8. The most sensitive strain *C. krusei* for CGA-N46 changed to be *C. tropicalis* for CGA-N15 and CGA-N12.

**Table 4:** MIC and hemolytic activity of CGA-N46 and its derived peptides

Peptide	MIC (mmol L^−1^)					5 % Hemolysis (mmol L^−1^)
	*C. glabrata*	*C. parapsilosis*	*C. krusei*	*C. tropicalis*	*C. albicans*	
CGA-N15	0.11	0.13	0.13	0.073	0.13	0.031
CGA-N16	0.28	0.32	0.38	0.41	0.38	0.056
CGA-N12	0.26	0.27	0.27	0.075	0.28	0.39
CGA-N8	0.3	0.3	0.24	0.29	0.24	0.27
CGA-N46	0.48	0.48	0.37	0.50	0.50	0.74

The hemolytic activity of CGA-N46 and its derived peptides against the highly sensitive human erythrocytes was determined as a measure of its toxicity to mammalian cells. The release of hemoglobin was monitored by measuring the absorbance at 570 nm. As negative and positive controls, erythrocytes in PBS without CGA-N46 and 0.1 % (v/v) Triton X-100 in PBS were employed, respectively. CGA-N46 and CGA-N12 showed weak hemolytic activity at the concentration of MIC. For CGA-N8, the MIC was near the concentration of its 5 % hemolysis. Meanwhile, CGA-N15 and CGA-N16 showed strong hemolytic activity. Comparing the ratio of the 5 % hemolytic concentration with the MIC for the sensitive yeasts, CGA-N12 (5.2-fold) is higher than that of CGA-N46 (twofold).

## Discussion

Andreu et al. [25] shortened an antimicrobial peptide with 26 amino acids without losing antimicrobial activity. Park et al. [26] found the antimicrobial buforin I C-terminal with 21 amino acids had stronger antimicrobial activity than parent peptides. In this study, we shortened CGA-N46 to find stronger antifungal-derived peptide with better biological characters based on the bioinformatics analysis and obtained derived fragments CGA-N16, CGA-N15, CGA-N12 and CGA-N8.

There were reports that some peptides including clavanin A formed random coil structure in aqueous solution [17, 24, 27, 28]. In contrast, peptides folded into certain secondary structures in the presence of trifluoroethanol [27, 28]. van Kan et al. [24] reported that the folding behavior of the peptides in aqueous solution highly depends on the nature of the amino acids. Different from the reports, CGA-N46 and its derivatives displayed α-helix in aqueous solution and SDS environments, but conformation changed in SUVs.

CGA-N46 and its derived peptides had inhibitory effects on the growth of fungi, but their antifungal activity was different. CGA-N12 was the inner sequence of CGA-N16, which was also the inner sequence of CGA-N46. They shared the same active center. The results of MTT assay suggested that the amount and features of amino acids influenced the antifungal activity of peptides.

As common structural characteristics, AMPs are peptides with net positive charges and adopt amphipathic structures [29]. Three kinds of models, including barrel–stave model, toroidal (or worm-hole) model and carpet model, have been proposed how AMPs finally form ion channels, transmembrane pores or extensive membrane rupture. The toroidal model appears to be more consistent with the mechanism of most AMPs [30]. The structure of the AMPs is necessary to lead the AMPs insert into the membrane. The bioinformatics analysis results of CGA-N46 and its derived peptides suggested that CGA-N46 and its derived peptides were weak positive charge or neutral, hydrophilic peptides. The results of CD demonstrated that the secondary structure of CGA-N46 and its derived peptides were α-helical in PBS and SDS, and they changed with α-helix decreasing and β-sheet, β-turn and random coil increasing when the solvents changed from PBS to SUVs. The results suggested that CGA-N46 and its derived peptides did not share the similar action mechanism with common α-helical AMPs.

As defenders in innate immunity, AMPs select ‘killing’ microbial without destroying human red cells [31]. Vasostatin-I was supposed to be one of innate immunity factors in mammalian [32]. Our study demonstrated that CGA-N12 showed strong antifungal activity and the least hemolytic activity.

## Conclusions

Based on the bioinformatics analysis of CGA-N46, a series of CGA-N46 derivatives were synthesized. The structure, antifungal activities, hemolytic activities were studied. Among the derivatives, CGA-N12 had higher antifungal activity and less hemolytic effect. It would be a potential candidate for further preclinical research.
